# The Thyroid Hormone Receptors Inhibit Hepatic Interleukin-6 Signaling During Endotoxemia

**DOI:** 10.1038/srep30990

**Published:** 2016-08-03

**Authors:** Constanza Contreras-Jurado, Elvira Alonso-Merino, Cristina Saiz-Ladera, Arturo José Valiño, Javier Regadera, Susana Alemany, Ana Aranda

**Affiliations:** 1 Departamento de Fisiopatología Endocrina y del Sistema Nervioso, Instituto de Investigaciones Biomédicas “Alberto Sols”, Consejo Superior de Investigaciones Científicas and Universidad Autónoma de Madrid, Madrid, Spain; 2 Departamento de Anatomía, Histología y Neurociencia, Facultad de Medicina, Universidad Autónoma de Madrid, Madrid, Spain

## Abstract

Decreased thyroidal hormone production is found during lipopolysaccharide (LPS)-induced endotoxic shock in animals as well as in critically ill patients. Here we studied the role of the thyroid hormone receptors (TRs) in activation of STAT3, NF-κB and ERK, which play a key role in the response to inflammatory cytokines during sepsis. TR knockout mice showed down-regulation of hepatic inflammatory mediators, including interleukin 6 (IL-6) in response to LPS. Paradoxically, STAT3 and ERK activity were higher, suggesting that TRs could act as endogenous repressors of these pathways. Furthermore, hyperthyroidism increased cytokine production and mortality in response to LPS, despite decreasing hepatic STAT3 and ERK activity. This suggested that TRs could directly repress the response of the cells to inflammatory mediators. Indeed, we found that the thyroid hormone T3 suppresses IL-6 signalling in macrophages and hepatocarcinoma cells, inhibiting STAT3 activation. Consequently, the hormone strongly antagonizes IL-6-stimulated gene transcription, reducing STAT3 recruitment and histone acetylation at IL-6 target promoters. In conclusion, TRs are potent regulators of inflammatory responses and immune homeostasis during sepsis. Reduced responses to IL-6 should serve as a negative feedback mechanism for preventing deleterious effects of excessive hormone signaling during infections.

Sepsis is characterized by an excessive inflammatory response to infection and is a major cause of mortality^1^. Endotoxic shock, the most lethal form of sepsis, is caused by lipopolysaccharide (LPS), the main membrane component of Gram-negative bacteria. The liver plays a critical role in innate and adaptive immunity. In response to infection and inflammation, the liver synthetizes acute-phase proteins (APPs), which are key components of the immune response to infection^2^,^3^. Innate immune responses triggered by LPS are mediated by Toll-like 4 receptors and involve the coordinated production of a large variety of inflammatory mediators, particularly Interleukin 6 (IL-6) and Tumor Necrosis α (TNFα)^4^. Activation of Signal Transducer and Activator of Transcription 3 (STAT3) and Nuclear Factor kappa-Light-chain-enhancer of Activated B Cells (NF-κB) by these cytokines plays a key role in the liver response to inflammation^3^, controlling the expression of a large number of genes^5^. IL-6 leads to activation of STAT3 and/or Ras–mitogen-activated protein kinase (MAPK) signaling^6^, while TNFα induces the activation of NF-κB^7^. In response to IL-6, STAT3 is phosphorylated at tyrosine 705^8^, triggering dimerization and STAT translocation into the nucleus, where it binds to its consensus motifs in target genes, including APP genes^2^,^9^. Activation of STAT3 also induces a negative feedback involving induction of phosphatases and Suppressor of Cytokine Signaling 3 (SOCS3)^10^. In non-stimulated cells, cytoplasmic NF-κB dimers are associated to inhibitory IκB proteins. In response to pro-inflammatory stimuli, such as TNFα, IκB is degraded, causing nuclear translocation and binding of NF-κB to its cognate sequences^11^. Some of the target genes are common for NF-κB and STAT3 and both transcription factors are engaged in positive and negative cross-talk^2^.

The actions of the thyroid hormones (L-thyroxine, T4, and 3,3′,5-triiodo-L-thyronine, T3) are mediated by binding to nuclear receptors (TRα and TRβ). These receptors act as ligand-dependent transcription factors by binding, generally as heterodimers with the retinoid X receptor (RXR), to thyroid hormone response elements in target genes or by modulating the activity of other transcription factors or signalling pathways^12^. There is increasing evidence that the thyroid hormones could modulate immune responses^13^,^14^, but the molecular mechanisms involved in the cross-talk between thyroid hormone and immune function have not yet been clarified. On the other hand, illness results in a strong reduction in thyroid hormone production and in changes in thyroid hormone metabolism, a condition called the ‘sick euthyroid syndrome’ or ‘nonthyroidal illness syndrome’ (NTIS)^15^. LPS administration serves as a model for NTIS in mice^16^. During sepsis, thyroidal T4 and T3 synthesis is down-regulated by cytokines^17^, expression of deiodinases responsible for thyroid hormone metabolism is altered^16^,^18^,^19^ and TRβ and RXR expression is reduced^20^,^21^.

We have previously shown that TRs can antagonize NF-κB activation in a ligand-dependent manner in pituitary cells^22^,^23^ and that they could modulate STAT3, NF-κB and ERK activation in the skin *in vivo*^24^. This suggests that TRs could regulate inflammatory responses. In this work we demonstrate that these receptors control hepatic cytokine production and signalling during sepsis *in vivo* and that the thyroid hormone directly antagonizes IL-6 signalling in cultured hepatocarcinoma cells and macrophages, leading to reduced STAT3 transcriptional activity.

## Results

### STAT3 and ERK over-activation in TR KO mice liver

**Figure 1:**
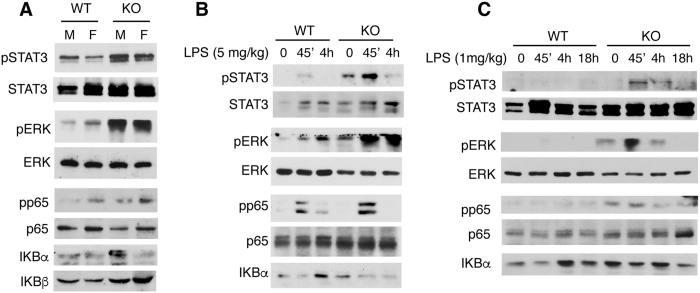
TRs deletion increases hepatic STAT3 and ERK activation. (**A**) Western blots with liver extracts of WT and TR KO mice and the indicated antibodies. Both male (M) and female (F) mice were used. (**B**) Protein levels assayed in male mice treated with vehicle or 5 mg/kg BW of LPS for the indicated time periods. (**C**) Similar experiment in WT and KO mice treated with 1 mg/kg BW LPS. Each band represents pooled samples from 3 different animals. Full-length blots are presented in Supplementary Figure 9.

To analyse whether TRs could modulate the activity of signalling pathways involved in immune responses, we used mice lacking TRα1 and TRβ, the main thyroid hormone binding isoforms^12^. These animals are extremely resistant to the actions of the thyroid hormones, presenting very high circulating levels of these hormones due to the lack of the feedback mechanism by which high thyroid hormone levels suppress pituitary thyroid-stimulating hormone^25^,^26^. Since liver inflammation and carcinogenesis show a clear gender disparity^27^, we measured the levels of total and phosphorylated STAT3 in male and female mice, finding that pSTAT3 levels were higher in the livers of KO animals of both sexes (Fig. 1A). Strongly increased ERK phosphorylation, without changes in total levels of the MAPK, and a weaker increase in p65/NF-κB phosphorylation with minor changes in total p65 and IκBs were also observed in TR KO mice.

The role of TRs in the liver response of these pathways to endotoxic shock was examined in male WT and TR KO mice sacrificed at 45 min or 4 h after injection of 5 mg/kg LPS (Fig. 1B). LPS treatment increased pSTAT3 and pERK levels and this induction was stronger in mice lacking TRs. LPS also enhanced p65 phosphorylation and caused a transient IκBα reduction, indicating NF-κB activation in both groups, although pp65 levels were slightly higher and IκBα levels were still low at 4 h in KO animals. This suggests that these signalling pathways could be could be more sensitive to LPS in TR KO mice. Therefore, we next examined the liver response to a lower LPS dose (1 mg/kg). This LPS concentration only induced significant phosphorylation of STAT3 and ERK in TR deficient mice, confirming that endogenous liver TRs exert a repressive effect in the activity of the examined pathways (Fig. 1C).

**Figure 2:**
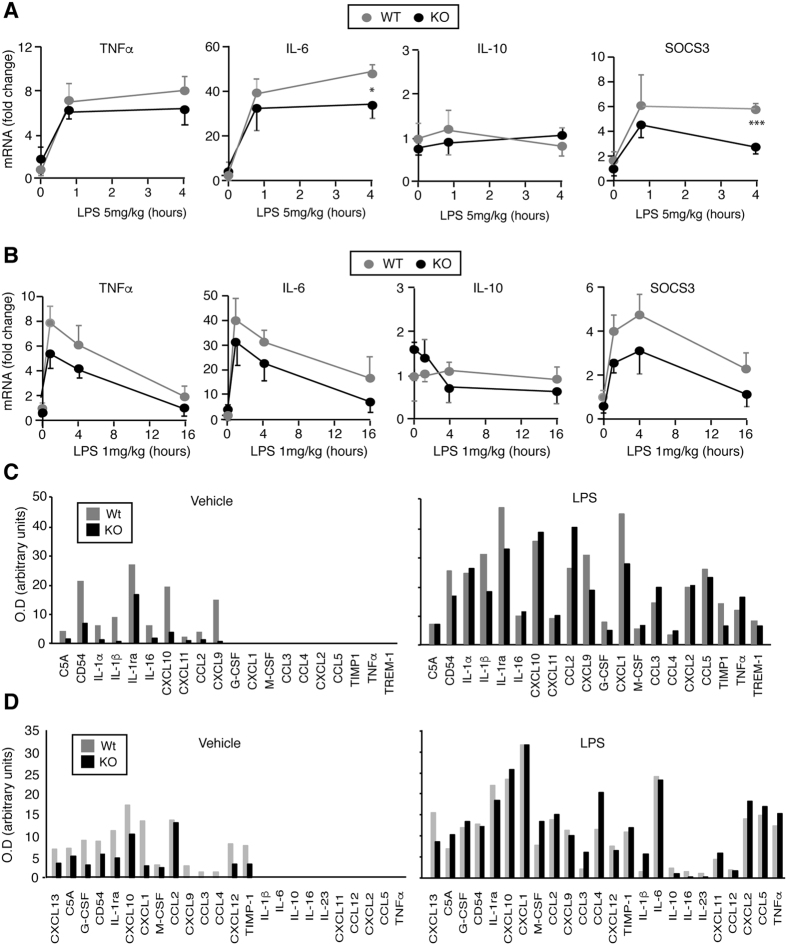
Cytokine expression in TR KO mice. (**A**) mRNA levels of *TNFα*, *IL-6*, *IL-10* and *SOCS3* in livers from WT and KO mice treated for 0 h, 45 min and 4 h with 5 mg/kg LPS. Data (means ± s.e) are expressed as fold-induction over the values obtained at time 0 in the untreated WT animals. Significance of Bonferroni post-hoc test (n = 3) between WT and KO mice is indicated. (**B**) Levels of the same mRNAs after injection of 1 mg/kg LPS for the indicated times. (**C**) Levels of cytokines and chemokines in livers from WT and KO mice determined using a Mouse Cytokine Array. Extracts from livers of animals treated with vehicle (left panel) or with 5 mg/kg LPS for 4 h (right panel) were pooled and used in the assay. (**D**) Circulating levels of cytokines and chemokines in the same groups.

Since these differences could be due to altered cytokine production and IL-6 and TNFα appear to be key components of the hepatic response to LPS, we measured transcript levels of these cytokines in the livers of WT and KO mice. No significant differences were found between untreated WT and KO animals and the same occurred with the anti-inflammatory *IL-10* cytokine or with *SOCS3* mRNAs. In addition, treatment with 5 mg/kg LPS (Fig. 2A) or with 1 mg/kg LPS (Fig. 2B) resulted in the expected increase of *TNFα* and *IL-6* transcripts, although *IL-6* transcripts were lower in KO mice after 4 h of treatment with the higher LPS concentration. This was unexpected, since STAT3 phosphorylation is increased in these animals and IL-6 appears to be a major contributor for STAT activation under these conditions. We next determined the hepatic levels of chemokines and proinflammatory cytokines after 4 h treatment with 5 mg/kg LPS using cytokine arrays. TNFα, IL-6 and IL-10 are barely detectable in the arrays, but other inflammatory mediators show clear changes between WT and KO mice (Fig. 2C and Supplementary Fig. S1A). Thus, the levels of Cluster of Differentiation 54 (CD54), several interleukins or CXCL9 and CXCL10 were reduced in the livers of untreated TR KO mice. Cytokine and chemokine expression was strongly stimulated by LPS, and under these conditions differences between both groups were less marked, although decreased expression of some proteins was still observed in KO mice. These results show that despite presenting increased STAT3 and ERK phosphorylation, TR deficient mice have reduced hepatic levels of chemokines and pro-inflammatory cytokines. Since macrophages and other immune cells are the major cytokine-producing cells, we also measured the levels of circulating inflammatory cytokines (Fig. 2D and Supplementary Fig. S1B). In concordance with liver data, serum levels of several cytokines and chemokines were reduced in the untreated KO mice. Again LPS induced a strong increase in the expression of many inflammatory mediators, and under these conditions no important differences between both groups were found. Of interest, serum levels of TNFα and IL-6 were strongly increased by LPS reaching similar levels in WT and KO animals.

### Thyroid hormone administration reduces STAT3 and ERK activation in response to LPS

**Figure 3:**
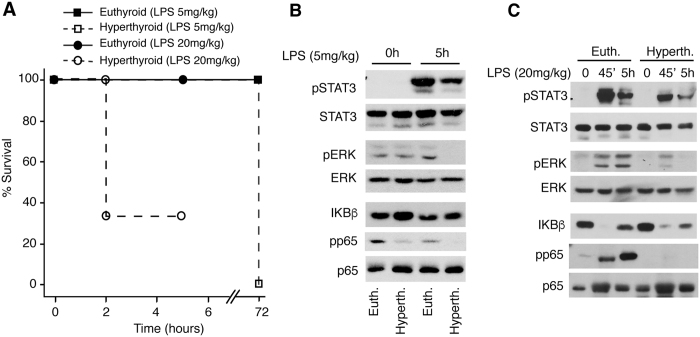
Reduced phosphorylation of STAT3, ERK and p65/NF-κB in hyperthyroid livers. (**A**) % survival of euthyroid and hyperthyroid male mice after treatment with 5 and 20 mg/kg LPS. (**B**) Western blots of the indicated proteins performed with liver extracts from control euthyroid (Euth.) and hyperthyroid (Hyperth.) mice. Mice were treated with vehicle or 5 mg/kg LPS for 5 h. Each band represents pooled extracts of 4–6 animals. (**C**) Similar experiment in euthyroid and hyperthyroid mice treated with 20 mg/kg LPS for 45 min and 5 h. Extracts from 4–6 animals were pooled except in the hyperthyroid group treated with LPS for 5 h, where only 2 mice survived. Full-length blots are presented in Supplementary Figure 9.

We next tested the possibility that excessive thyroid hormone signalling could be associated with the opposite phenotype to that found in TR KO mice. For this purpose, the response to LPS was analysed in euthyroid mice and in thyroid hormone treated mice. mRNA levels of Diodinase 1 (*Dio* 1), an accurate marker of hepatic thyroid hormone action were significantly higher in the thyroid hormone treated mice demonstrating tissue hyperthyroidism (Supplementary Fig. S2A,B). Mice were treated with vehicle or LPS at two different doses, 5 mg/kg or 20 mg/kg BW. Of note, hyperthyroidism severely decreased survival during sepsis. Four out of six of the thyroid hormone-treated mice died within 2 h of injection with 20 mg/kg LPS, while all control animals appeared healthy and survived the treatment for at least 5 h. With the lower LPS dose, all euthyroid animals survived for 72 h, but hyperthyroid mice showed 100% mortality at this time (Fig. 3A). Therefore, only short-term experiments were performed to analyse the effect of hyperthyroidism in pathway activation and cytokine production in response to LPS. Opposite to the results obtained in TR-deficient mice, hyperthyroid livers showed reduced STAT3 and ERK phosphorylation in response to administration to either 5 mg/kg LPS (Fig. 3B) or 20 mg/kg LPS (Fig. 3C). NF-κB activation was again demonstrated by rapid disappearance of IκBα, which almost returned to normal levels after 5 h. Also in contrast with the TR-deficient mice, p65 phosphorylation was lower in the untreated hyperthyroid mice and they showed a strongly reduced response to LPS treatment. Therefore, these signalling pathways appear to be altered in an opposite way by TR-deficiency and by thyroid hormone excess.

**Figure 4:**
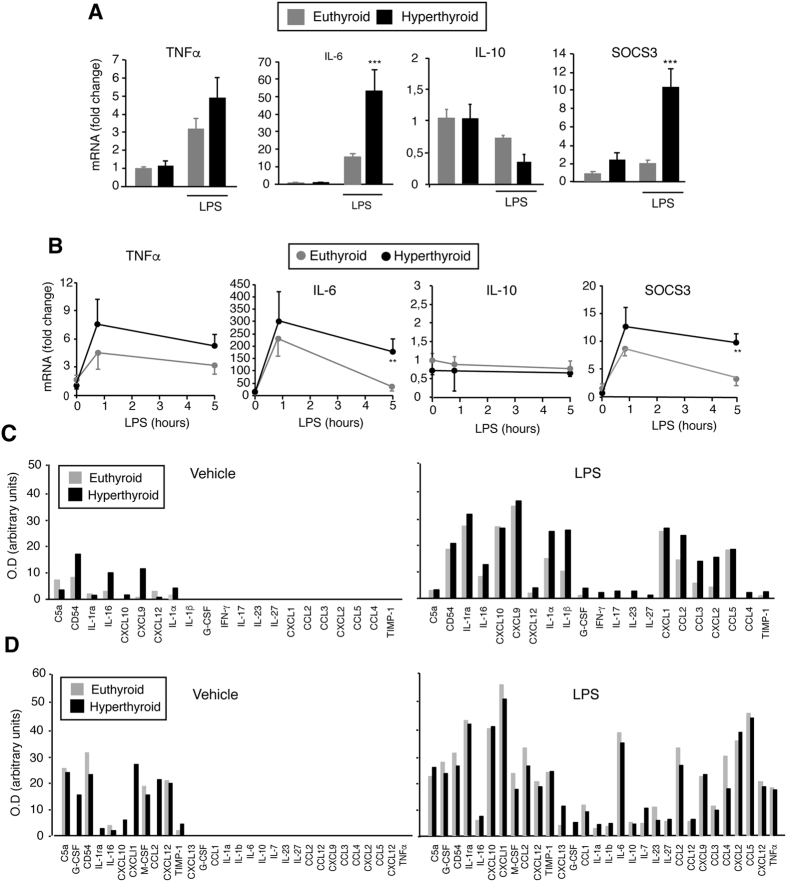
Cytokine levels in the liver and serum of hyperthyroid mice. (**A**) Levels of the indicated transcripts in livers from euthyroid and hyperthyroid mice treated for 0 and 5 h with 5 mg/kg LPS or vehicle. Data (means ± s.e of 4–6 mice) are expressed as fold-induction over the values obtained at time 0 in the untreated controls. Significance of post-hoc ANOVA test between euthyroid and hyperthyroid animals is indicated with asterisks. (**B**) Levels of the same mRNAs after treatment with 20 mg/kg LPS for 0 h, 45 min and 5 h. (**C**) Quantification of cytokine and chemokine arrays in pooled livers from mice treated with vehicle (left panel) or with 5 mg/kg LPS for 5 h (right panel). (**D**) Circulating levels of cytokines and chemokines in the same animals.

Also opposite to the results obtained in TRs KO mice, hyperthyroidism resulted in increased hepatic levels of *IL-6* and *SOCS3* transcripts in response to 5 mg/kg LPS (Fig. 4A) or 20 mg/kg LPS (Fig. 4B), while *TNFα* and *IL-10* mRNAs remained unchanged. Cytokine profiling showed that thyroid hormone treatment increased basal expression of CD54, IL-16, CXCL9 or CXCL10 that were reduced in the KO mice (Fig. 4C and Supplementary Fig. 3A). Again, LPS treatment (5 mg/kg for 5 h) strongly induced the levels of different chemokines and cytokines. Under these conditions differences between both groups were attenuated, although increased levels of some of these immune mediators were still observed in hyperthyroid mice. Serum levels of several proteins including CXCL10, CXCL11, CCL2 or G-CSF were also increased in hyperthyroid mice before LPS treatment (Fig. 4D and Supplementary Fig. S3B), although most of the differences between control and hyperthyroid animals again disappeared after the treatment. Despite difference of *IL-6* mRNA expression in the liver, circulating IL-6 levels were similarly induced by LPS in both groups.

### Increased macrophage infiltration in hyperthyroid mice

To analyse if the observed changes in cytokine production and pathways activation were reflected in altered liver damage or infiltration of inflammatory cells, liver histology was performed in TR-deficient and hyperthyroid animals and their corresponding controls before and after treatment with LPS (5 mg/kg for 4–5 h). AST activity was not still enhanced at this early time (Supplementary Figs S4A and S5A), but LPS treatment caused a detectable increase of the number of inflammatory cells inside the blood vessels in all groups. In all hyperthyroid animals, but not in other groups, sporadic necrotic foci with infiltrating macrophages were found after LPS-treatment (Supplementary Figs S4B and S5B,C). Furthermore, no liver damage that could explain early lethality in hyperthyroid mice treated with 20 mg/kg LPS was observed, discarding liver failure as responsible for death (Supplementary Fig. S6).

### T3 reduces Il-6 signalling in cultured cells

**Figure 5:**
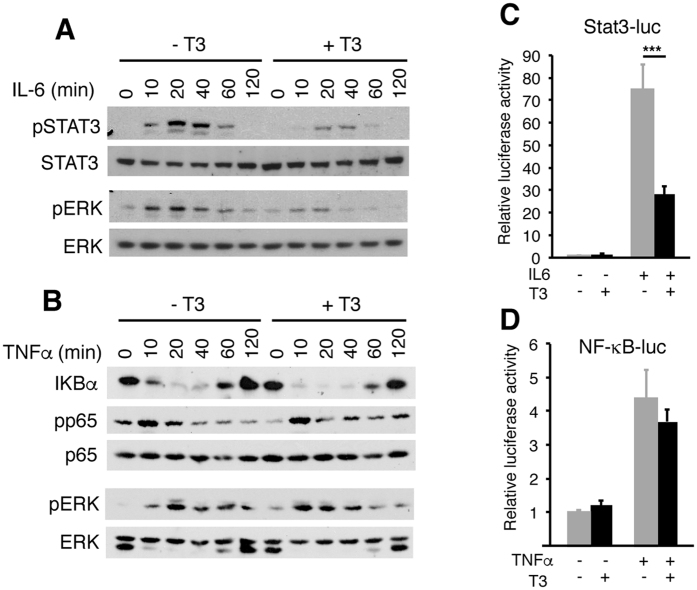
T3 antagonizes IL-6 signaling in Hep3B cells. (**A**) Proteins were extracted from Hep3B cells pretreated with 5 nM T3 in 10% thyroid hormone-depleted serum for 36 h and then treated with 10 ng/ml IL-6 for times ranging from 0 to 120 min. The levels of phosphorylated and total STAT3 and ERK were assessed by Western blot. (**B**) Western blot analysis of the indicated proteins in cells pretreated with T3 and then incubated with 10 ng/ml TNFα for varying times. Full-length blots are presented in Supplementary Fig. 9. (**C**) Cells were transiently transfected with a reporter plasmid containing STAT binding sites and incubated in the presence and absence of T3 for 36 h and with IL-6 for the last 5 h. (**D**) Luciferase activity in cells transfected with a reporter plasmid containing NF-kB binding sites and treated with T3 for 36 h and/or TNFα for the last 5 h.

The discrepancy between cytokine production and pathway activation observed *in vivo*, suggested that the thyroid hormones could directly regulate cytokine signalling in hepatic cells. Therefore, we next tested STAT3 and ERK activation by IL-6 in cultured Hep3B cells (Fig. 5A). Phosphorylation of STAT3 and ERK was strongly and transiently induced by IL-6, but this induction was markedly blunted in T3-treated cells showing that the hormone inhibits IL-6 mediated activation of both signalling pathways. Similar results were obtained in cells cultured with 10% serum (Fig. 5A) and 0.5% serum (Supplementary Fig. S7). As expected, IL-6 did not cause IκB degradation or p65 phosphorylation illustrating that it does not activate NF-κB (Supplementary Fig. S7). To analyse the effect of T3 on NF-κB activation cells were treated with TNFα. TNFα did not stimulate STAT3 phosphorylation, but caused ERK activation, a rapid and transient disappearance of IKB and a detectable phosphorylation of p65 that were not significantly altered by T3 (Fig. 5B). The lack of inhibitory effect of T3 on the NF-κB response to TNFα was confirmed under low serum conditions (Supplementary Fig. S7).

**Figure 6:**
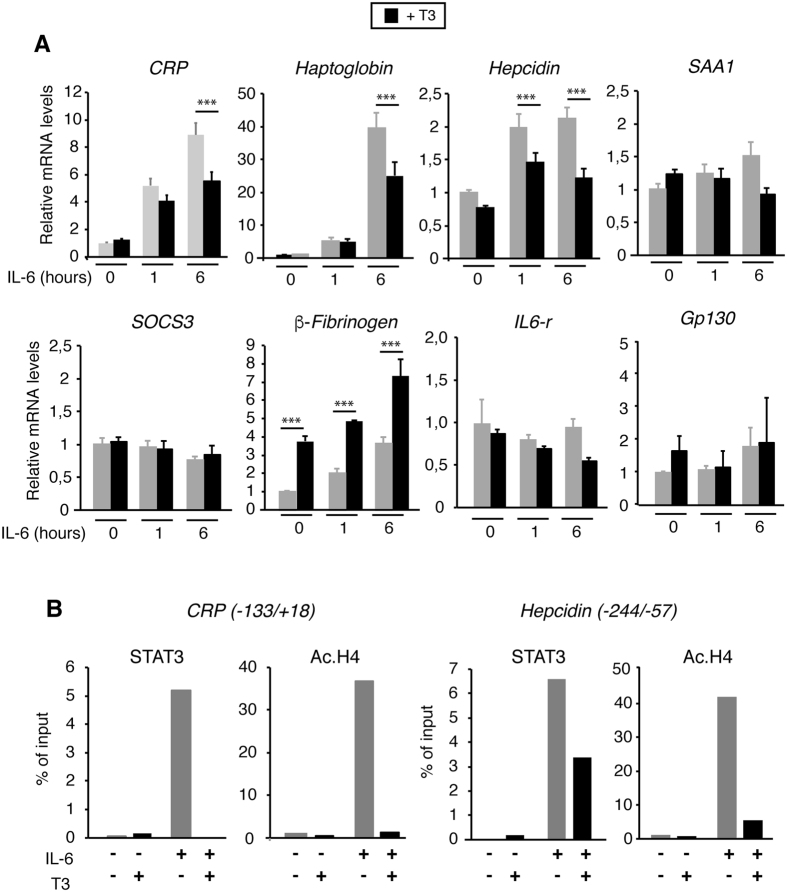
T3 represses transcriptional activation of STAT3-dependent genes by IL-6. (**A**) Levels of the indicated transcripts (means ± s.d) determined in Hep3B cells treated with 5 nM T3 for 36 h and with IL-6 for 0, 1 and 6 h. Significance of post-hoc ANOVA test between cells treated with and without T3 is indicated. (**B**) ChIP assays with the indicated regions of the *CRP* and *Hepcidin* genes and antibodies against STAT3 or acetylated H4 (Ac.H4). Data are expressed of % of the input after subtracting the values obtained with a control IgG. One representative experiment out of 2 with similar results is shown.

To test the effect of T3 in STAT3-dependent transcriptional activity, we performed transient transfection assays with a reporter plasmid bearing STAT-binding elements in Hep3B cells. IL-6 markedly stimulated the activity of the reporter, and T3 strongly antagonized this response (Fig. 5C). In contrast, the hormone did not reduce significantly activation by TNFα of a reporter plasmid containing NF-κB binding sites (Fig. 5D). Since several genes encoding APPs contain STAT-responsive sites in their regulatory regions^2^,^9^, we next examined the effect of T3 in IL-6 mediated stimulation of APP transcript levels. As shown in Fig. 6A, IL-6 caused a significant increase on *C-Reactive Protein* (CRP), *Haptoglobin* and *Hepcidin* mRNA levels that was strongly antagonized in T3-treated cells. In Hep3B cells the expression of the *Serum Amiloid A*-1 (*SAA1*) or *SOCS3* genes was not responsive to IL-6 and consequently T3 did not alter their levels. In addition, transcripts of the IL6 receptor or Glycoprotein 130 (*Gp130*), the common subunit of the type I cytokine receptor, essential for IL-6 signal transduction were not altered by T3. Finally, and in agreement with previous results β–*Fibrinogen* gene expression was stimulated by T3^28^ and also by IL-6.

To evaluate STAT3 recruitment to target promoters *in vivo*, ChIP assays were performed with chromatin from Hep3B cells treated with IL-6 and/or T3 (Fig. 6B). Un-stimulated cells did not show significant STAT3 binding to the *CRP* or *Hepcidin* promoter regions containing STAT3 response elements^2^,^3^. However, STAT3 was recruited to the promoters in IL-6-treated cells, and T3 significantly reduced this response in agreement with the repression of endogenous gene activation. The abundance of acetylated histone H4, a marker for transcriptional activation, followed a similar pattern since T3 also suppressed IL-6 dependent increase of histone acetylation (Fig. 6B).

**Figure 7:**
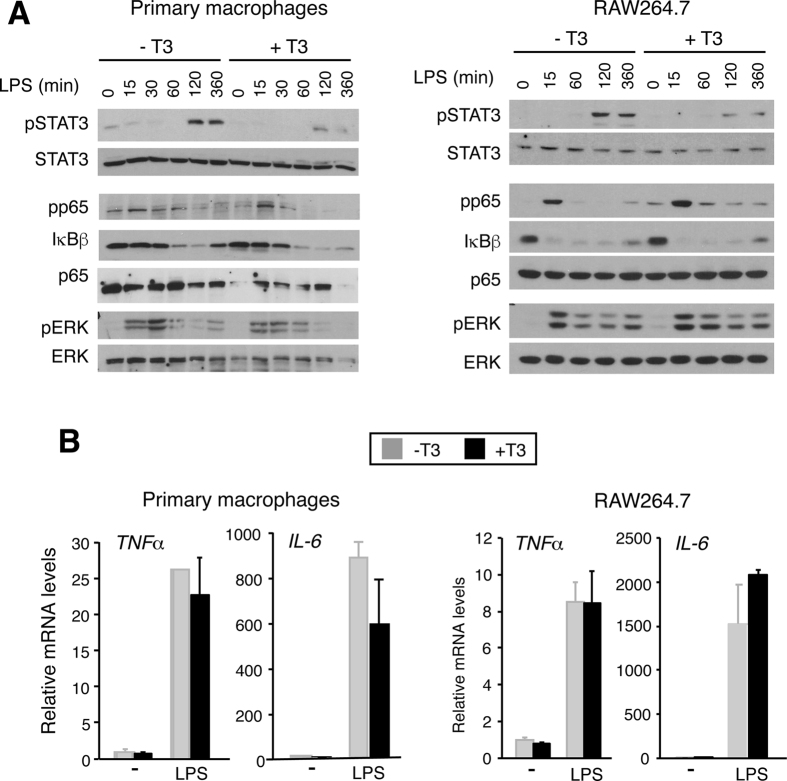
T3 reduces STAT3 activation in macrophages. (**A**) Western blot analysis of the indicated total and phosphorylated proteins in primary cultures of bone marrow macrophages (left) and RAW264.7 macrophages (right) pretreated with 5 nM T3 in 10% thyroid hormone-depleted serum for 36 h and then treated with 300 ng/ml LPS for the indicated time points. Full-length blots are presented in Supplementary Figure 9. (**A**) Levels of *TNFα* and *IL-6* mRNA (means ± s.d) in bone marrow and RAW264.7 macrophages treated with T3 for 36 h and with LPS for the last 6 h.

As macrophages are major cytokine-producing cells, playing a key role in the response to endotoxemia, we also examined how the hormone affected LPS, IL-6 or TNFα signalling in these cells. T3 strongly reduced LPS-induced STAT3 phosphorylation in primary cultures of macrophages and in the murine RAW264.7 macrophage cell line, while having minor effects in ERK or NF-κB activation (Fig. 7A). This reduction was not due to a lower cytokine production in response to the endotoxin, since *TNFα* and *IL-6* mRNA induction by LPS was similar in control and T3-treated cells (Fig. 7B). T3 also markedly reduced pSTAT3 levels in response to IL-6, but did not alter the NF-κB response to TNFα in RAW264.7 macrophages (Supplementary Fig. S8). Therefore, T3 is also a strong antagonist of IL-6 signalling in macrophages.

## Discussion

Here we report a novel role for TRs and their ligands, the thyroid hormones, in the activation of signalling pathways that play an essential role in liver homeostasis and inflammation. STAT3 phosphorylation, which mediates dimerization, nuclear translocation and transcriptional stimulation^8^, was increased in the livers of genetically modified mice lacking TRs. Hepatic ERK phosphorylation was also enhanced and a modest increase of p65 phosphorylation was also detected. Augmented activation of these signalling molecules was also found after LPS treatment, the archetypical inflammatory stimulus causing sepsis and endotoxic shock. In view of these results, our initial working hypothesis was that TRs could play a repressor role in the production of cytokines and chemokines that are crucial in the recruitment of inflammatory cells and that are in turn prominent amongst STAT3, ERK and NF-κB targets are genes^29^. Surprisingly, induction of IL-6, the main cytokine responsible for hepatic STAT3 activation during sepsis, was reduced in TR deficient mice. An increase in circulating IL-6 that could reach the hepatic cells and account for the increased STAT3 activation was also dismissed. Although STAT3 is predominantly activated by IL-6, other cytokines such as IL-10 also activate this transcription factor^30^,^31^. However, no changes in IL-10 were observed and therefore increased STAT activity in TR-deficient livers cannot be attributed to increased production of this cytokine. Furthermore, when hepatic expression of an array of cytokines and chemokines, as well as their circulating levels, was measured it was found that their levels were generally lower in TR-deficient mice. These results indicate that increased STAT3 and ERK phosphorylation detected in these animals in response to LPS is most definitely not due to increased cytokine production, and they emphasize the important role of these receptors as endogenous repressors of these signalling pathways since they are overactive even in the presence of abnormally low levels of cytokines. Intriguingly, we have previously shown that increased cytokine content is found in the skin of TR-deficient mice in response to treatment with 12-O-Tetradecanoylphorbol-13-acetate^24^. Therefore, the effect of TRs on cytokine production and inflammatory responses is a complex phenomenon that appears to differ depending on the tissue and type of inflammatory insult.

Excessive thyroid hormone signalling led in general to opposite hepatic changes to those observed in the TR KO mice. Increased production of several cytokines and chemokines was found in the liver and serum of thyroid hormone-treated animals. However, circulating levels of most of these proteins reached similar levels in euthyroid and hyperthyroid mice after endotoxic shock, probably reflecting that extrahepatic cytokine production is not greatly influenced by thyroid hormone treatment. Under these conditions, hepatic expression of the *IL-6* gene is more sustained in LPS treated hyperthyroid mice, despite presenting similar circulating levels of this cytokine. Again, there was a clear dissociation between the levels of inflammatory mediators and hepatic STAT3, ERK or NF-κB activity, as phosphorylation of these signalling molecules was not increased but rather significantly reduced in hyperthyroid livers.

Although the LPS dosages used were well tolerated by all control mice, thyroid hormone-treated mice showed increased lethality, indicating that they are more sensitive to the endotoxic shock caused by cytokine hyper-production. TNFα production is believed to account for the fatal shock syndrome, which includes a prominent hepatic component^32^. Interestingly, lethality was associated with the specific up-regulated hepatic expression of IL-6, but not TNF-α in hyperthyroid mice. However, no changes in the hyperthyroid livers that could explain early lethality were found and therefore an extra-hepatic mechanism that remains to be defined should be responsible for the reduced survival to endotoxic shock. Although STAT3 activation in hepatocytes could attenuate lethality in sepsis^33^, hypersensitivity to LPS induced endotoxic shock has been related to increased IL-6 production^34^. Therefore, reduced STAT3 activation might be involved in the increased mortality in hyperthyroid mice and a reduced response of the hepatic and/or extra-hepatic cells to this cytokine could represent a defence mechanism by which these animals could try to constrain sepsis-induced mortality. This is in line with the observation that thyroid hormone production by the thyroid gland is reduced not only after LPS administration in mice^16^ but also in NTIS in humans^15^. Interestingly, IL-6 appears to be involved in the development of this syndrome, since the drop in circulating thyroid hormones is reduced in IL-6 KO mice^35^.

The dissociated effects of the thyroid hormones in cytokine levels and the activation status of their signalling pathways suggested that these hormones could act directly in the liver cells or in other cells to repress cytokine signalling. We demonstrate that indeed liganded TRs directly interfere with inflammation-based cell signalling pathways in cultured Hep3B cells. T3 suppresses IL-6 signalling by inhibiting the activation of the main downstream targets of the cytokine: STAT3 and ERK. This was not due to reduced expression of the IL-6 receptors and therefore TRs appear to inhibit STAT3 activation downstream of binding of IL-6 to its cognate receptors. Furthermore, STAT3 phosphorylation in response to LPS was reduced by T3 in primary cultures of macrophages, as well as in the murine RAW264.7 macrophage cell line, although the hormone did not reduce the induction of *IL-6* gene expression by the cytokine. STAT3 activation in response to incubation with IL-6 was also suppressed by T3 in macrophages, indicating that inhibition of IL-6 signalling by the thyroid hormones also occurs in these cells that have potent regulatory functions during infection and inflammation.

IL-6 is known to be a strong stimulator of APP gene expression in liver cells^9^. In addition to T3 being a counter-regulator of IL-6 stimulated STAT3 phosphorylation, the hormone also antagonized induction of APP genes in response to the cytokine, reducing STAT3 binding to their regulatory regions. Stimulation of transcription by STAT factors involves the recruitment of coactivators with histone acetyltransferase activity^36^, and T3 also decreased the abundance of acetylated histone H4, a mark for transcriptional activation. Some of the examined genes such as *SAA1* or even *SOCS3* that contain STAT response elements and are well-known targets of this cytokine were not responsive to IL-6 in Hep3B cells, indicating the importance of the cell context and the limitations of the cultured cell models. In any case, our results suggest that the thyroid hormone could counteract the response of liver cells to the cytokine excess produced *in vivo* under hyperthyroid conditions by reducing its transcriptional actions and serving as a negative feedback defence mechanism. Besides this mechanism, inhibition of thyroidal secretion^16^, as well as the reduced expression of TR and its heterodimeric partner RXR^20^,^21^ should contribute to limit the deleterious effects of excess thyroid hormone signalling during LPS-induced sepsis.

The changes in p65 phosphorylation found in the livers of TR KO and hyperthyroid mice suggested that the liganded receptors could also antagonize NF-κB activation that plays a crucial role in the response to pro-inflammatory cytokines. We have previously shown that T3 impairs NF-κB activation by TNFα or by serum deprivation in pituitary tumour cells, causing a significant decrease of NF-kB-dependent transcription^22^,^23^. However, T3 did not reduce NF-kB activation by LPS in macrophages or by TNFα in Hep3B cells. In pituitary cells this inhibition involves the induction of the MAPK phosphatase, Dual Specificity Phosphatase 1. It is possible that this mechanism does not operate in macrophages or Hep3B cells. Therefore, more studies will be needed to clarify a possible antagonistic effect of TRs in NF-kB-activation in the liver *in vivo*.

The liver is a central organ in maintaining metabolic homeostasis and in controlling inflammatory responses during infection. The known functions of TRs in the liver have expanded from their well-known roles in regulating liver metabolism^37^, to also participate in liver regeneration^26^, senescence^38^, fibrosis^39^ and hepatocarcinogenesis^40^. In this study we show that these receptors are also potent regulators of immune homeostasis during sepsis, showing a link between thyroid hormone signalling and STAT3 and ERK activation. Therefore, TRs may represent a confluence point of metabolic and inflammatory responses in the liver, suggesting that they could be important targets for developing new therapeutic strategies for the treatment of liver diseases. New thyromimetic drugs are being developed with the expectation that they could have beneficial effects in dyslipidemia, non-alcoholic fatty liver disease or even fibrosis. Our present results showing increased mortality to sepsis in thyroid hormone treated animals stress the importance of discovering novel ligands that could dissociate their beneficial metabolic effects from their potential danger in case of infections.

## Methods

### Animals and Treatments

All animal work was carried out in compliance with the European Community Law (86/609/EEC) and the Spanish law (R.D. 1201/2005), with approval of the Ethics Committee of the Consejo Superior de Investigaciones Científicas. Experiments were performed in livers from adult TRα1^−/−^/TRβ^−/−^ double knockout (KO) mice and in wild-type TRα1^+/+^/TRβ^+/+^ animals in a hybrid genetic background of 129/OLa+129/Sv+ BALB/c+C57BL/6. In addition, 8 weeks old wild-type male C57BL/6J mice bred at the Instituto de Investigaciones Biomédicas were made hyperthyroid by adding T4 (25 ng/g of mice) and T3 (95 ng/g of mice) in the drinking water during 15 days. Animals were injected i.p with the LPS (at concentrations varying from 1 to 20 mg/kg BW) from *Escherichia coli* serotype 026:B6 Sigma–Aldrich, San Luis, MO) and sacrificed at different times. Serum was collected and livers samples were either frozen to obtain RNA and proteins or fixed with paraformaldehyde and embedded in paraffin.

### Protein extraction and western-blotting

Livers were homogenized and total proteins were extracted in Ripa Buffer (50 mM Hepes pH 7.5, 150 mM NaCl, 10% Glicerol, 1 mM EGTA, 1% Triton-X100, 1% Sodium Doxicolate, 0,1% SDS). Cells were harvested and lysed in triple-detergent lysis buffer (50 mM Tris-HCl pH 8.0, 150 mM NaCl, 0.1% SDS, 1% NP-40, 0.5% sodium deoxycholate). In all cases, phosphatase and protease inhibitor cocktail tablets (Roche, Basel, Switzerland) were added. Protein lysates were mixed with Laemmli sample buffer, boiled and loaded onto 8% or 12% sodium dodecyl sulfate-polyacrylamide gel electrophoresis. Western analysis was performed using the antibodies and dilutions included in the Antibody Table.

### Cytokine and Chemokine Quantification

R&D Systems Mouse Cytokine Array, Panel A (Catalog # ARY006, Minneapolis, MN), was used to simultaneously detect the levels of 40 different cytokines and chemokines in 100 μl serum or 400 μg of whole-cell protein extracts obtained from livers from 3–4 animals per group and following the manufacturer specifications. Signals were detected by chemiluminiscence and were quantitated by scanning the X-ray films after different exposures (between 1 and 6 min) with ImageJ.

### AST activity

Serum AST activity was measured in 32 μl of a 1:3 dilution of sera with the Reflotron test (Roche), following the manufacturer’s instructions.

### Real-time quantitative RT-PCR (qRT-PCR)

RNA extraction, reverse transcription and quantitative PCR were performed as described previously^38^. The sequences of the oligonucleotides used in this study are listed in the Supporting Information.

### Histology and Immunohistochemistry

Liver samples were fixed in 4% buffered formalin and embedded in paraffin. Deparaffinized tissue sections were stained with H&E using standard procedures and images were obtained using a high-resolution Leica DC200 digital camera mounted on an Olympus DMLB microscope. Immunohistochemistry of the macrophage marker F4/80, a kind gift of Dr. Antonio Castrillo, was performed on 4 μm deparaffinized-rehydrated sections with 1:100 antibody dilution. Antigen retrieval was carried out with citrate buffer and endogenous peroxidase activity was inhibited with 3% H_2_O_2_. Samples were blocked and incubated overnight with the antibody, and signal was amplified with the ABC *Kit* (Vectastin). Slides were revealed with DAB (Vector), counterstained with Hematoxylin and mounted with DePeX (Serva).

### Cell culture

Hep-3B cells were grown in DMEM and RAW264.7 cells in RPMI with 10% FCS. Before the experiments cells were transferred to medium containing serum depleted of thyroid hormones by treatment with AG1-X8 resin. Bone marrow macrophages were obtained from the tibia and femur of 8 weeks-old C57-BL6 mice, plated and grown as previously described in DMEM and 30% medium conditioned by L929 fibroblasts^41^. After 6 days the medium was changed to DMEM containing 10% resin-treated serum and treatments started.

### Transient transfections and luciferase assays

Hep-3B cells were plated in DMEM with 10% FCS in 24-well plates. After 24 h, cells were transfected in serum free medium with 300 ng of luciferase reporter plasmids containing consensus NF-kB and STAT3 binding sites^23^,^42^ and 50 ng of pRL-TK-Renilla (Promega, San Luis, CA) as a normalizer control, using Lipofectamine 2000 (InVitrogen, Carlsbad, CA). After 6 h cells were transferred to medium supplemented with 0.5% thyroid hormone-stripped serum by treatment with AG1-X8 (Bio-Rad, Hercules, CA) resin. Cells were incubated with 5 nM T3 for 36 h in the presence and absence of IL-6 (10 ng/ml) or TNFα (10 ng/ml) for the last 5 h. Luciferase activity was determined with the Dual Luciferase Assay System (Promega). Experiments were performed in triplicate and repeated three times. Data are mean ± s.d and are expressed as fold induction over the values obtained in the untreated cells.

### ChIP assays

Hep-3B cells were plated in 150 mm dishes and after 24 h were washed and treated with 5 nM T3 for 36 h and/or 10 ng/ml IL6 for 2 h in medium containing 0.5% thyroid hormone-depleted serum. Cells were fixed and lysed following specifications of 17–295 Upstate kit, and sonicated in a *Bioruptor UCD-200TM* (Diagenode, Liège, Belgium). In each immunoprecipitation approximately 3 × 10^6^ cells and 5 μg of STAT3 antibody (sc-482(c-20)x), 1 μg of acetylated histone H4 antibody (Upstate 06-866) or 3 μg of normal rabbit IgG (sc-2027) were used. DNA was purified and precipitated. The promoter region of the C-Reactive Protein (*CRP*) promoter was amplified with the oligonucleotides 5′-GAAATAATTTTGCTTCCCCTCTTCCC-3′ (forward) and 5′-TCCTAGATCTCTTGCCTTAGAGCTACCTCC-3′ (reverse), and the promoter of the *Hepcidine* gene with the oligonucleotides 5′-CCCCACCCCCTGAACACA-3′ (forward) and 5′-ACCGAGTGACAGTCGCTTTT-3′ (reverse). Results were normalized and are presented as a fraction of the inputs after subtracting the values obtained with the control IgG, which was always below 1% of the input chromatin.

### Statistical analysis

Statistics were calculated with the SPSS software. Statistical significance of data was determined by applying ANOVA followed by the Bonferroni post- test. Results shown in the figures are means ± s.e. in experiments with animals or means ± s.d. in the experiments performed in cultured cells. *P*-values between WT and KO mice, euthyroid and hyperthyroid mice or cells treated with and without T3 are represented in the figures with asterisks: *P < 0.05, **P < 0.01 and ***P < 0.001.

## Additional Information

**How to cite this article**: Contreras-Jurado, C. *et al*. The Thyroid Hormone Receptors Inhibit Hepatic Interleukin-6 Signaling During Endotoxemia. *Sci. Rep.* **6**, 30990; doi: 10.1038/srep30990 (2016).

## Supplementary Material

Supplementary Information
