# Supplementary material for: The Thyroid Hormone Receptors Inhibit Hepatic Interleukin-6 Signaling During Endotoxemia
Source: Sci Rep. 2016 Aug 3;6:30990. doi: 10.1038/srep30990 (PMC4971531; doi:10.1038/srep30990)

Supplementary data

The Thyroid Hormone Receptors Inhibit Hepatic Interleukin-6 Signaling During Endotoxemia

Constanza Contreras-Jurado[1]*, Elvira Alonso-Merino[1]*, Cristina Saiz-Ladera[1], Arturo José. Valiño[1], Javier Regadera[2], Susana Alemany[1] and Ana Aranda[1].

[1]Departamento de Fisiopatología Endocrina y del Sistema Nervioso, Instituto de Investigaciones Biomédicas "Alberto Sols". Consejo Superior de Investigaciones Científicas and Universidad Autónoma de Madrid. [2] Departamento de Anatomía, Histología y Neurociencia, Facultad de Medicina, Universidad Autónoma de Madrid. Madrid, Spain.

Supplementary Materials and Methods

Antibodies used

Antibody	Isotype	Dilution	Catalog number
Anti-P-STAT3Tyr^{705}	Rabbit	1:1000	Cell Signaling 9145s
Anti-STAT3	Rabbit	1:1000	Cell Signaling C7907
Anti- P-ERK	Mouse	1:1000	Santa Cruz, sc-7383
Anti-ERK2	Rabbit	1:1000	Santa Cruz, sc-154
Anti-P-p65	Rabbit	1:500	Cell Signaling 3033
Anti-p65	Mouse	1:1000	Cell Signaling 6956P
Anti-IKBα	Rabbit	1:500	Santa Cruz, sc-371
Anti-IKBβ	Rabbit	1:500	Santa Cruz, sc-945

List of primers used for real-time quantitative RT-PCR (qRT-PCR)

	Amplification primers<br><br>Sequence (5´-3´)	
GENE	Forward	Reverse
Mouse IL6	GCTACCAAACTGGATATAATCAGGA	CCAGGTAGCTATGGTACTCCAGA
Mouse IL10	TCCTCCTCCAGCTCTTACCTC	TGGCTTTCCCTAGGACTCTCT
Mouse TNFα	CGATCACCCCGAAGTTCAGTA	GGTGCCTATGTCTCAGCCTCTT
Mouse Socs3	ATTTTCGCTTCGGGACTAGC	AACTTGCTGTGGGTGACCAT
Mouse GAPDH	TTCACACCATGGAGAAGGC	CCCTTTTGGCTCCACCCT
Mouse Dio1	GTTGAACTTTGGCAGTTGCAC	GGCTGTGGAGGCAAAGTCATC
Human Gp130	TCAACTTGGAGCCAGATTCC	CCCACTTGCTTCTTCACTCC
Human Il6-r	TTGTTTGTGAGTGGGGTCCT	TGGGACTCCTGGGAATACTG
Human CRP	CCCTGAACTTTCAGCCGAATACA	CGTCCTGCTGCCAGTGATACA
Human Haptoglobin	TTGCAGTGGACTCAGGCAAT	CAGCCGTCATCTGCTTCACAT
Human Hepcidin	CCCCACCCCCTGAACACA	ACCGAGTGACAGTCGCTTTT
Human SAA1	CTGCAGAAGTGATCAGCG	ATTGTGTACCCTCTCCCC
Human Socs3	ATCCTGGTGACATGCTCCTC	GGCACCAGGTAGACTTTGGA
Human β-fibrinogen	AGCAGCTGCCACTCAAAAGA	GAGGAGGTCTGGGAAACAGC

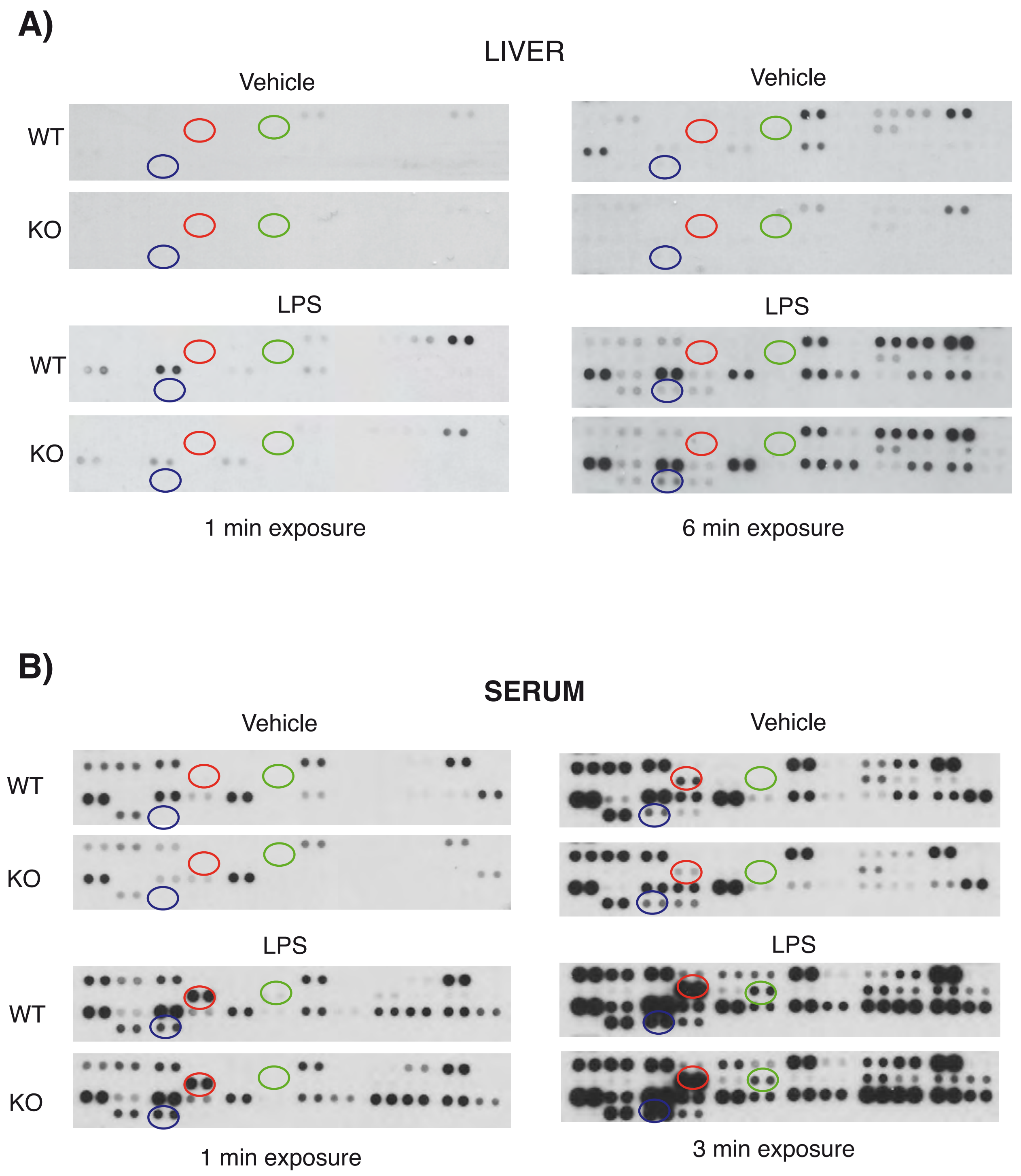

Fig. S1. TR deficiency decreases hepatic and circulating levels of several cytokines and chemokines. A) Mouse Cytokine Arrays using pooled liver extracts from WT and TR KO mice (3-4 animals/point) treated with vehicle or with LPS (5mg/kg) for 4h as indicated. To better visualize the reduction in the intensity of the spots in the KO group two different exposures of the arrays (1 min and 6 min) are shown. B) Arrays exposed for 1 min or 3 min obtained from pooled serum samples of the same animals. The blue, red and green circles show the position of the duplicate spots corresponding to TNFα, IL-6 and IL-10, respectively.

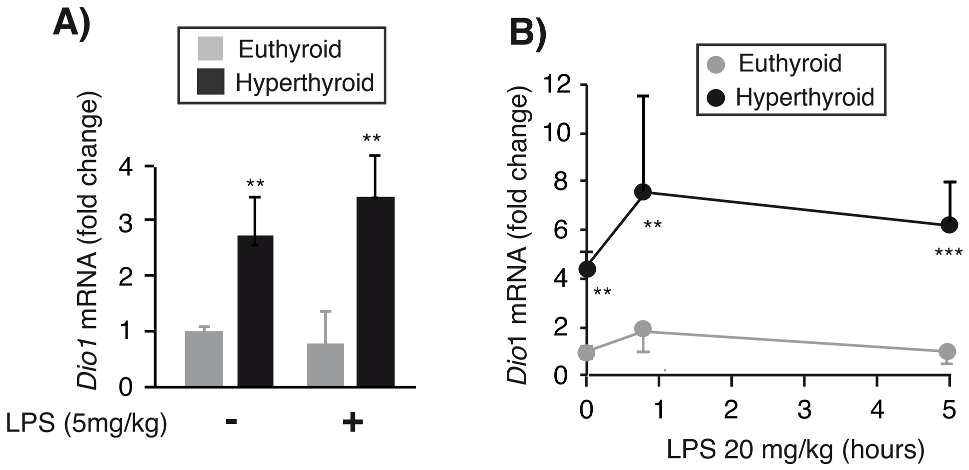

Fig. S2. Thyroid hormone treatment increases liver *Dio1* mRNA levels. A) To prove that oral thyroid hormone treatment was sufficient to induce hyperthyroidism, *Dio1* transcripts were measured in livers from euthyroid and hyperthyroid mice untreated and treated with vehicle or LPS (5mg/kg) for 5h. Data (means± s.e) are expressed relative to the values obtained in untreated euthyroid controls. Statistically significant differences between euthyroid and hyperthyroid mice are shown with asterisks. B) Similar experiments performed in mice injected with LPS (20mg/kg) for the indicated time periods.

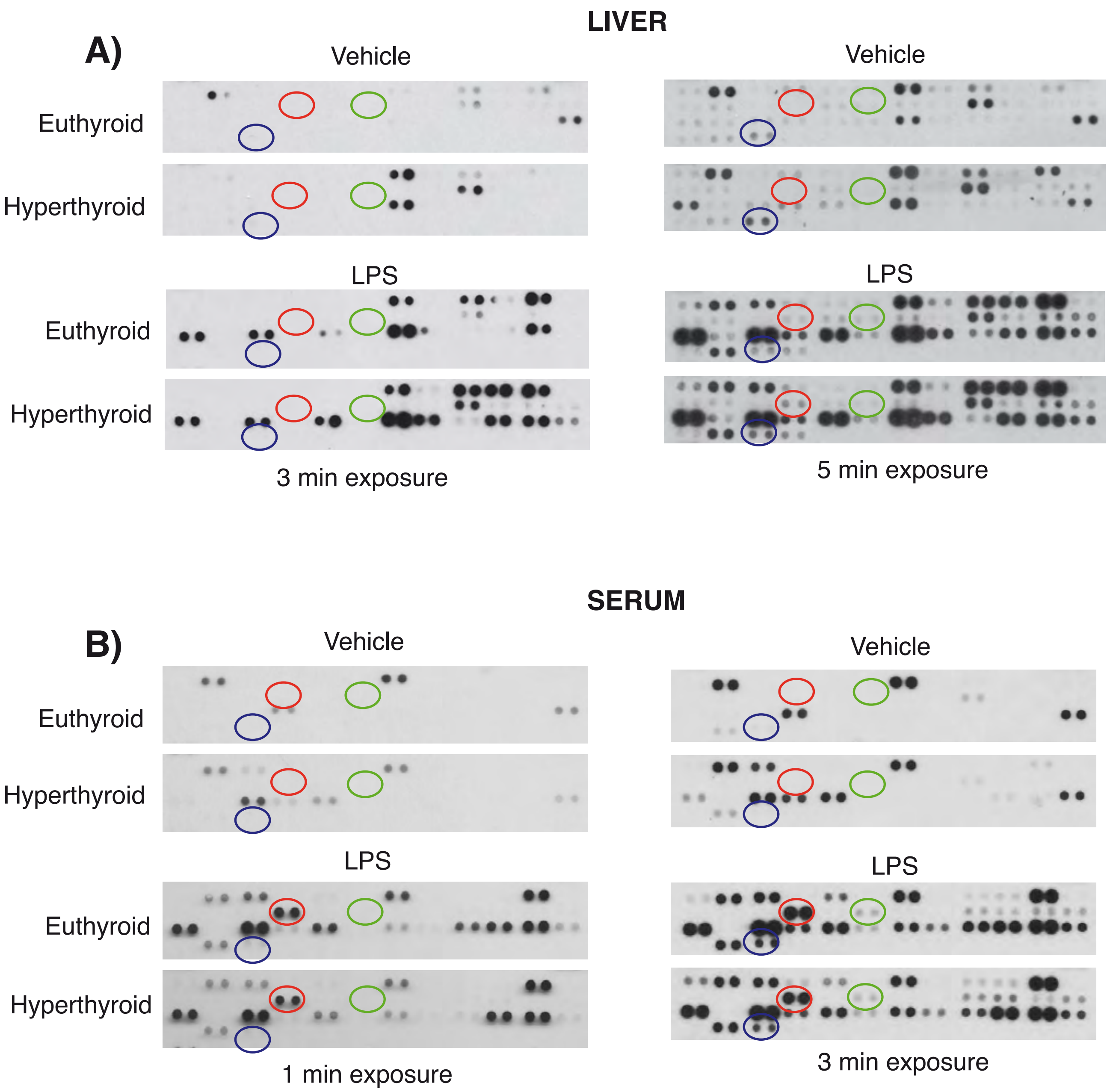

Fig. S3. Hepatic and circulating cytokines and chemokines in hyperthyroid mice. A) Liver extracts from euthyroid and hyperthyroid mice (pools from 4-6 animals) were used to detect cytokines and chemokines with Mouse Cytokine Arrays. Mice were treated with vehicle or with LPS (5mg/kg) for 5h. Two different exposures of the arrays (3 min and 5 min) are shown. The blue, red and green circles show location of the spots corresponding to TNFα, IL-6 and IL-10, respectively. Note that IL-6 is only clearly detectable at high exposures in hyperthyroid LPS-treated mice corresponding with the high IL-6 mRNA levels in the livers of these animals. B) Serum levels of cytokines and chemokines in the same animals. Arrays were exposed for 1 min and 3 min.

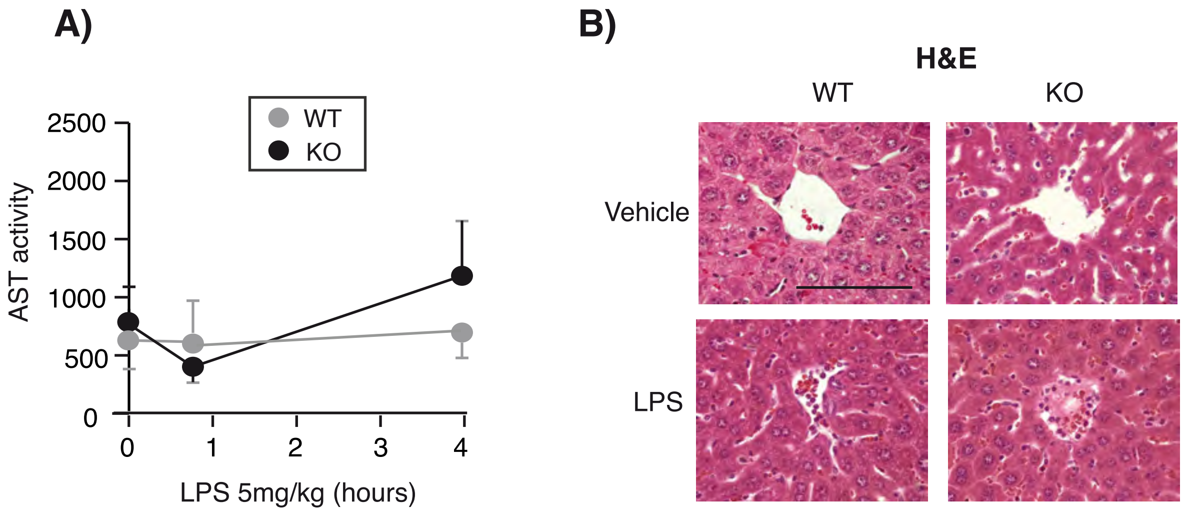

Fig. S4. Liver histology after LPS treatment of WT and TR KO mice. A) AST activity in serum of WT and TR KO mice treated with 5mg/kg LPS for the times indicated. B) Representative H&E staining of livers from mice treated with vehicle or LPS for 4h, showing an increased number of inflammatory cells within the vessels and absence of necrosis or inflammatory cell infiltration in the liver parenchyma. Scale bar: 100µm.

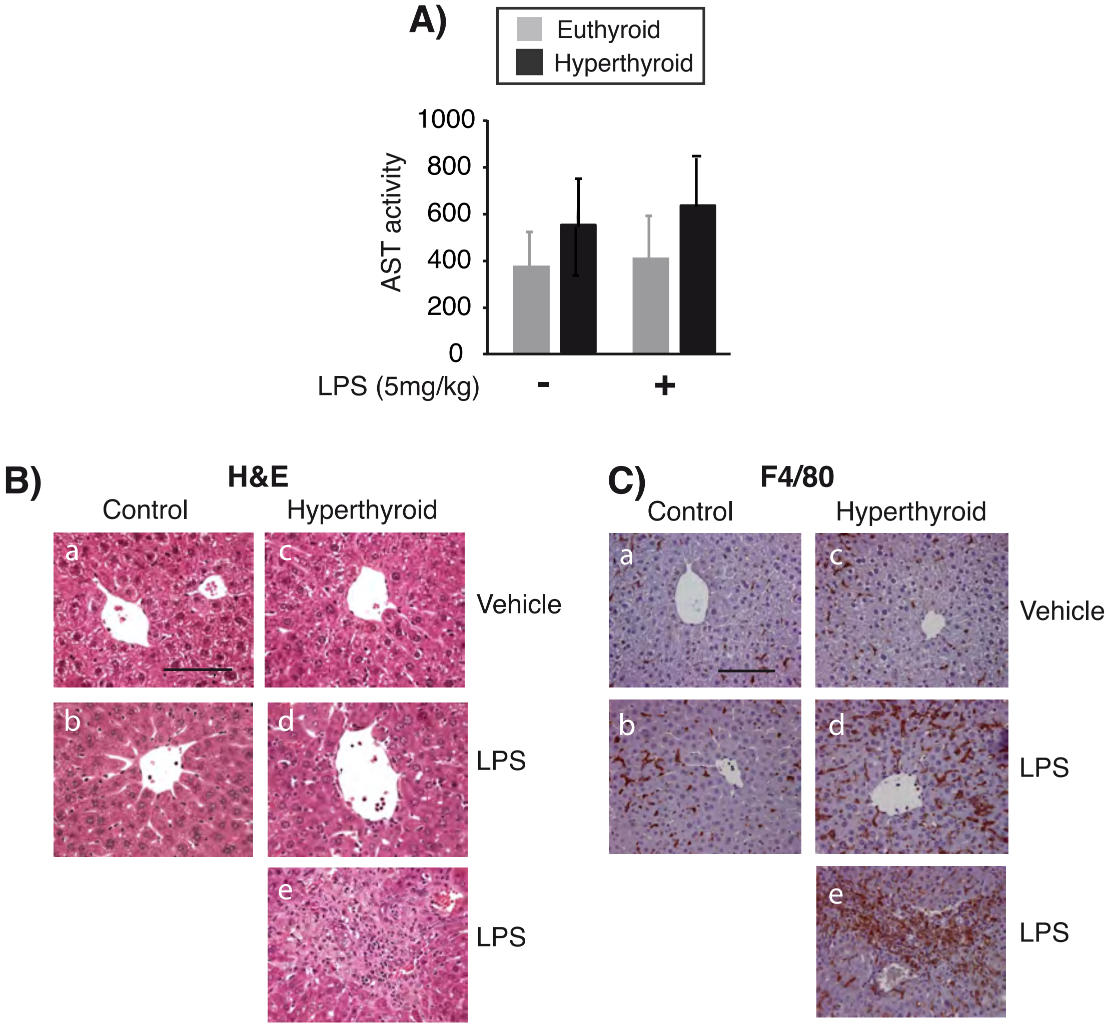

Fig. S5. Effect of hyperthyroidism in hepatic damage and immune cell infiltration in response to LPS. A) AST activity in serum of control and hyperthyroid mice treated with vehicle or 5mg/kg LPS for 5h. B) H&E staining of the livers of the different groups. Panel e shows the occasional appearance of necrotic foci with nuclei loss and inflammatory cell infiltration in LPS-treated hyperthyroid mice. Necrosis was not detected in the LPS-treated control mice. C) Immunohistochemistry of macrophages labeled with the F4/80 antibody in livers from the same animals, showing increased number of macrophages in hyperthyroid mice after LPS treatment. Panel e shows macrophage infiltration in the necrotic areas, which did not occur in euthyroid mice. Scale bars: 100µm.

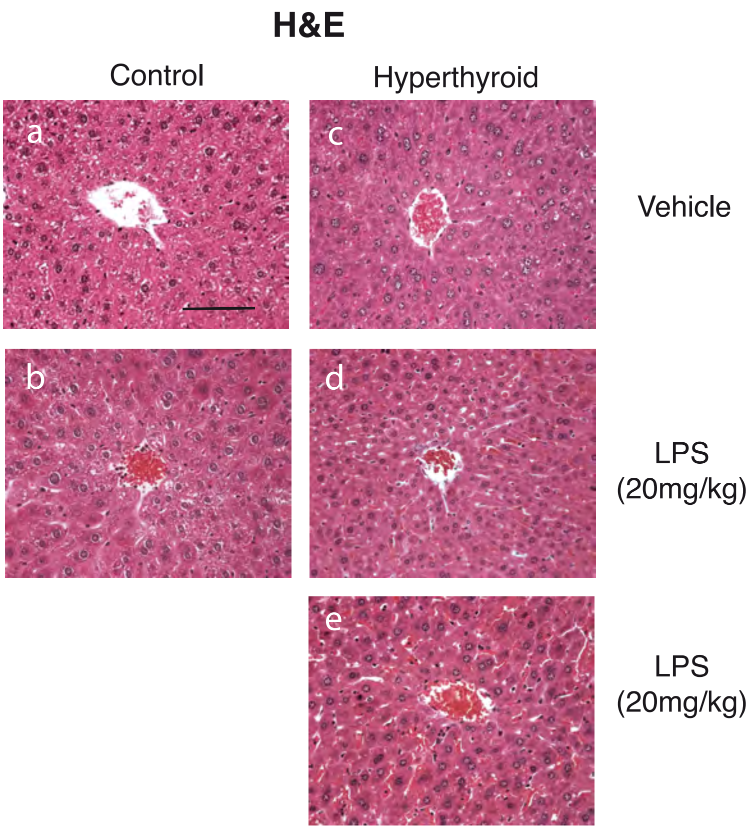

Fig. S6. H&E staining of livers from euthyroid and hyperthyroid mice treated with vehicle or 20 mg/kg LPS for 5h. Panel e shows liver morphology post-mortem in a representative mice that died 2 h after injection, showing absence of liver damage. Scale bar: 100µm.

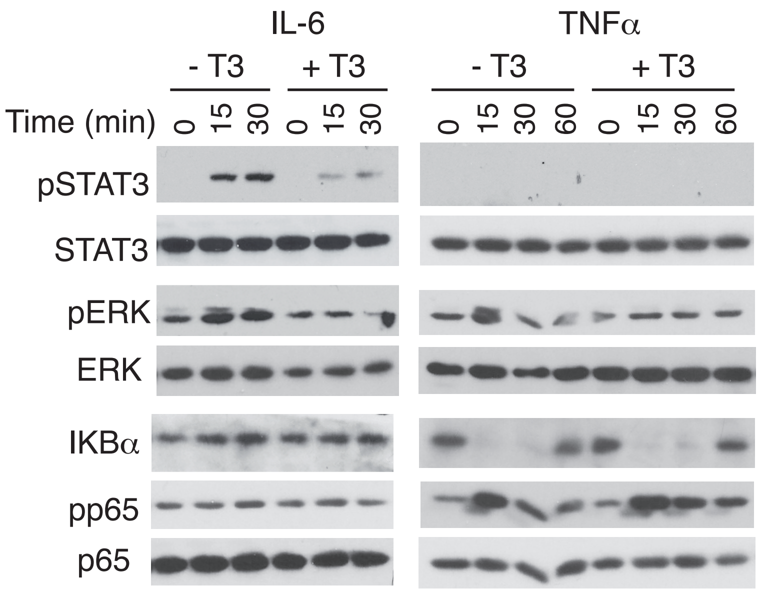

Fig. S7. T3 reduces signaling by IL-6 but not by TNFα in Hep3B cells. Western blots of tthe indicated proteins performed in cells treated with 5 nM T3 for 36 h and with IL-6 (10ng/ml) or TNFα (10ng/ml) for the indicated time periods in medium containing 0.5% thyroid hormone depleted serum.

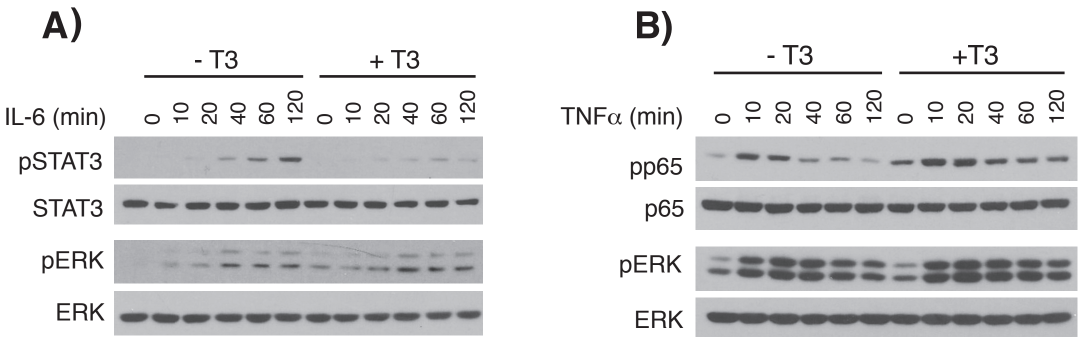

Fig.S8. T3 inhibits signaling by IL-6 but not by TNFα in macrophages. RAW264.7 cells were treated with 5nM T3 for 36h before addition of 10ng/ml IL-6 (A) or 10 ng/ml TNFα (B) for times varying between 0 and 120 min in medium containing 10% thyroid hormone depleted serum. The levels of the indicated total and phosphorylated proteins were analyzed by western blot.

Fig. S9 (blots)

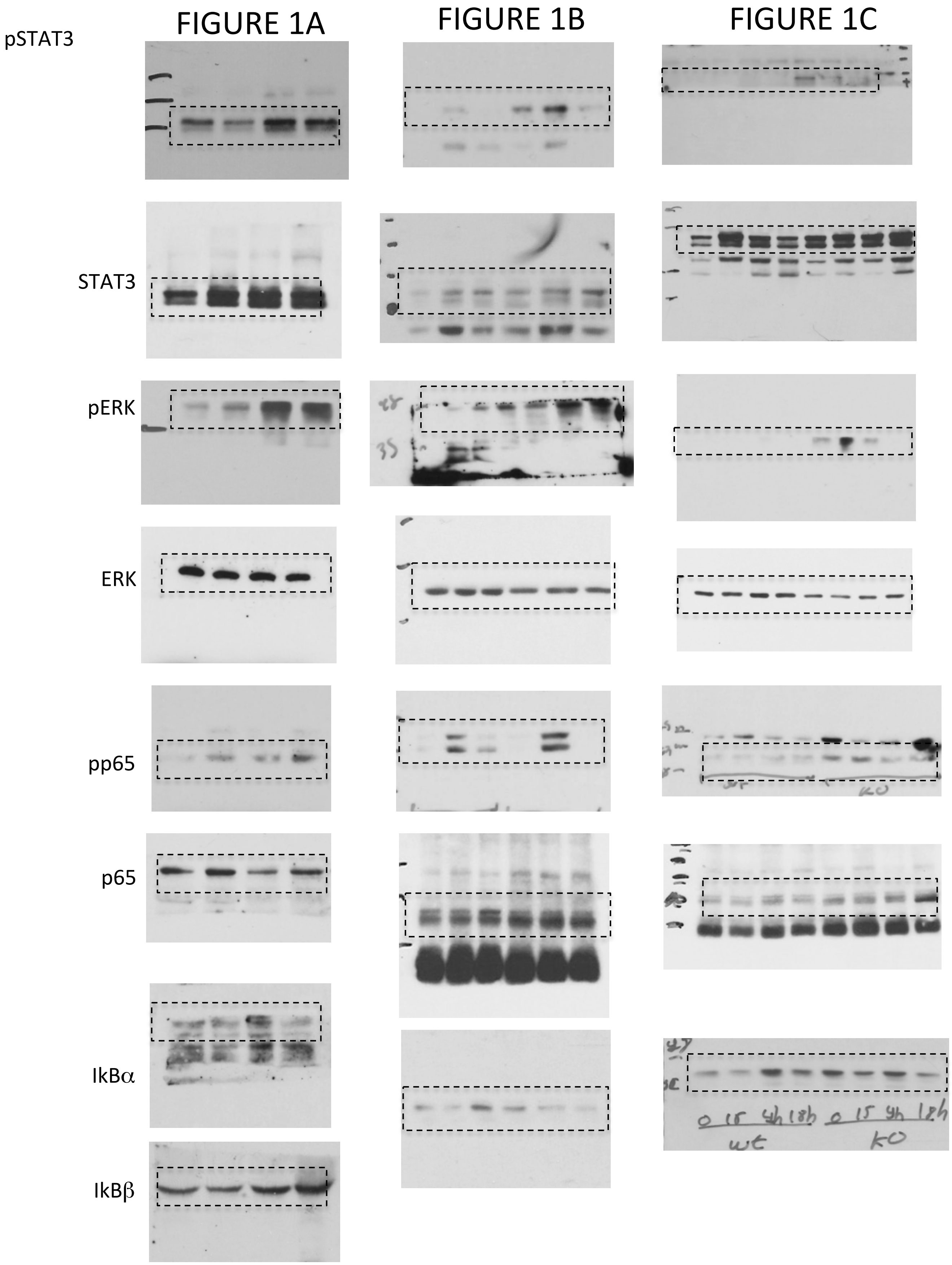
FIGURE 1A
FIGURE 1B
FIGURE 1C
pSTAT3
STAT3
pERK
ERK
pp65
p65
IkBα
IkBβ
0 15 4h 18h 0 15 4h 18h
wt
KO

FIGURE 3B

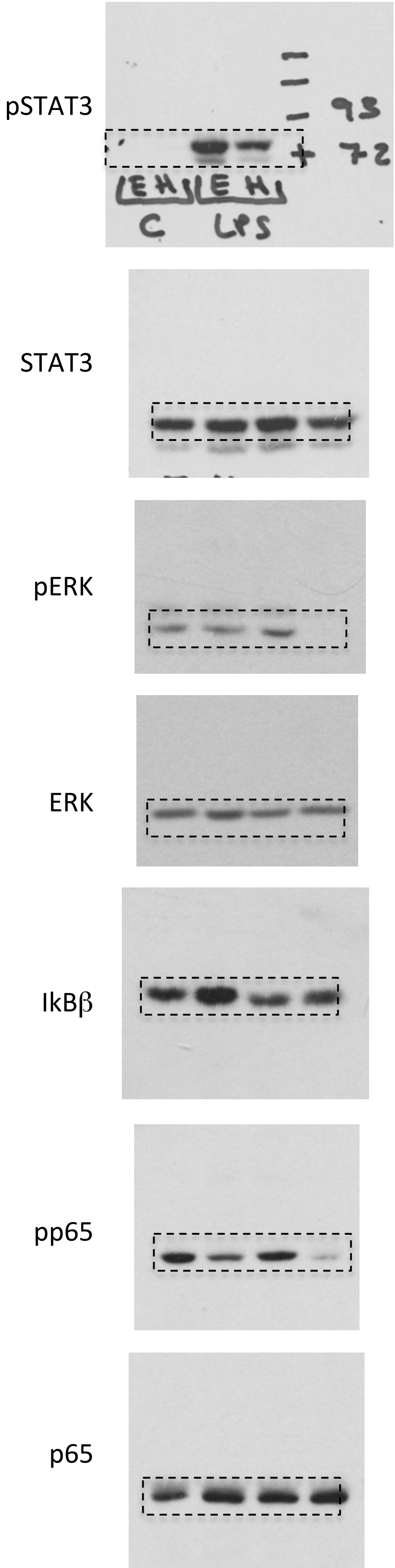

FIGURE 3C

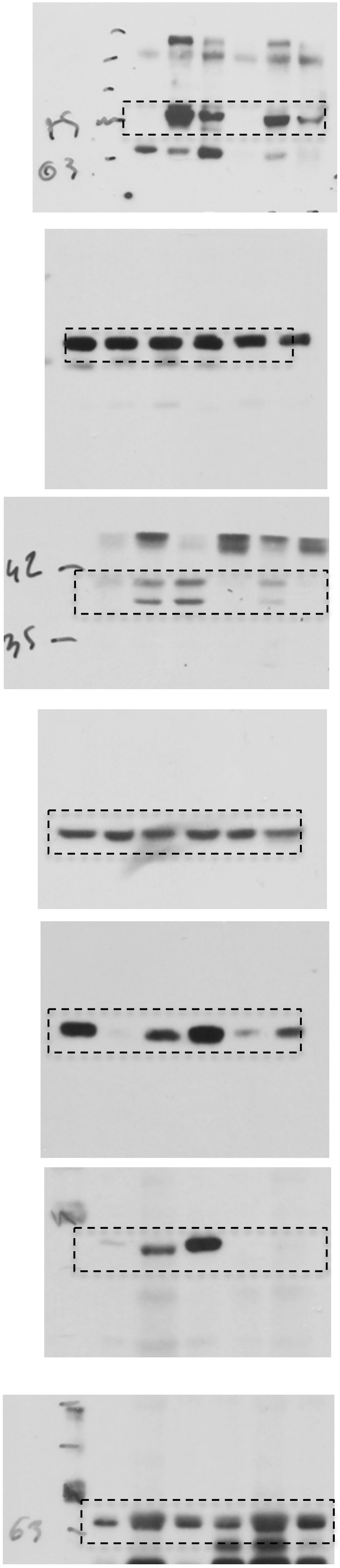

FIGURE 5A

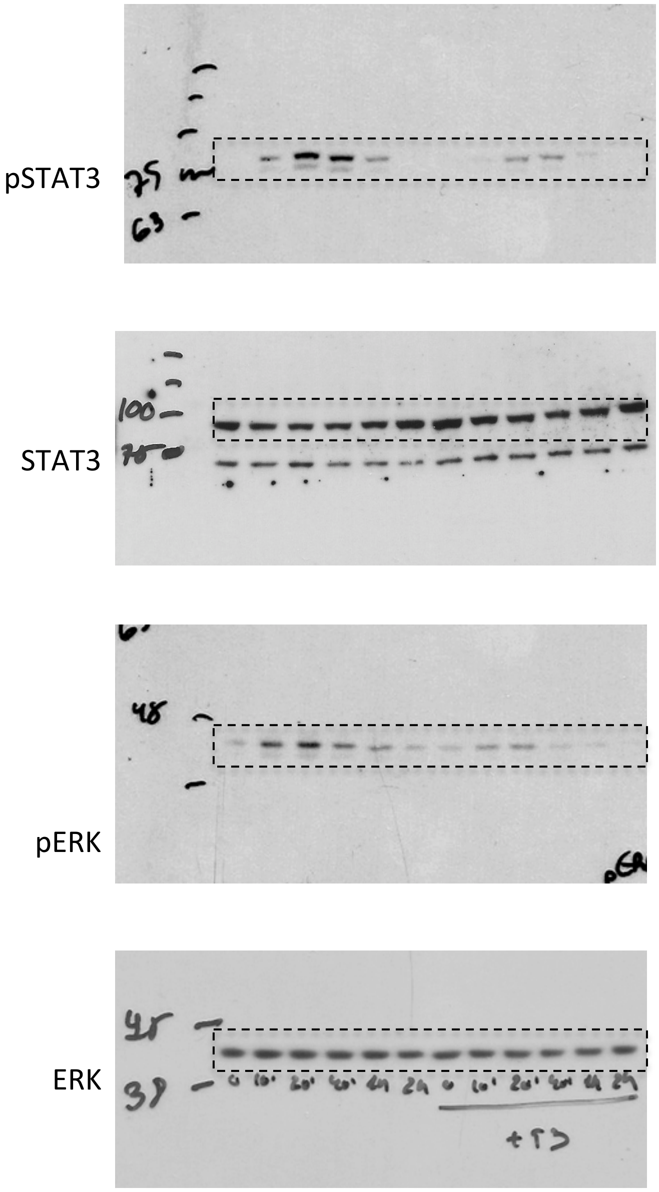

FIGURE 5B

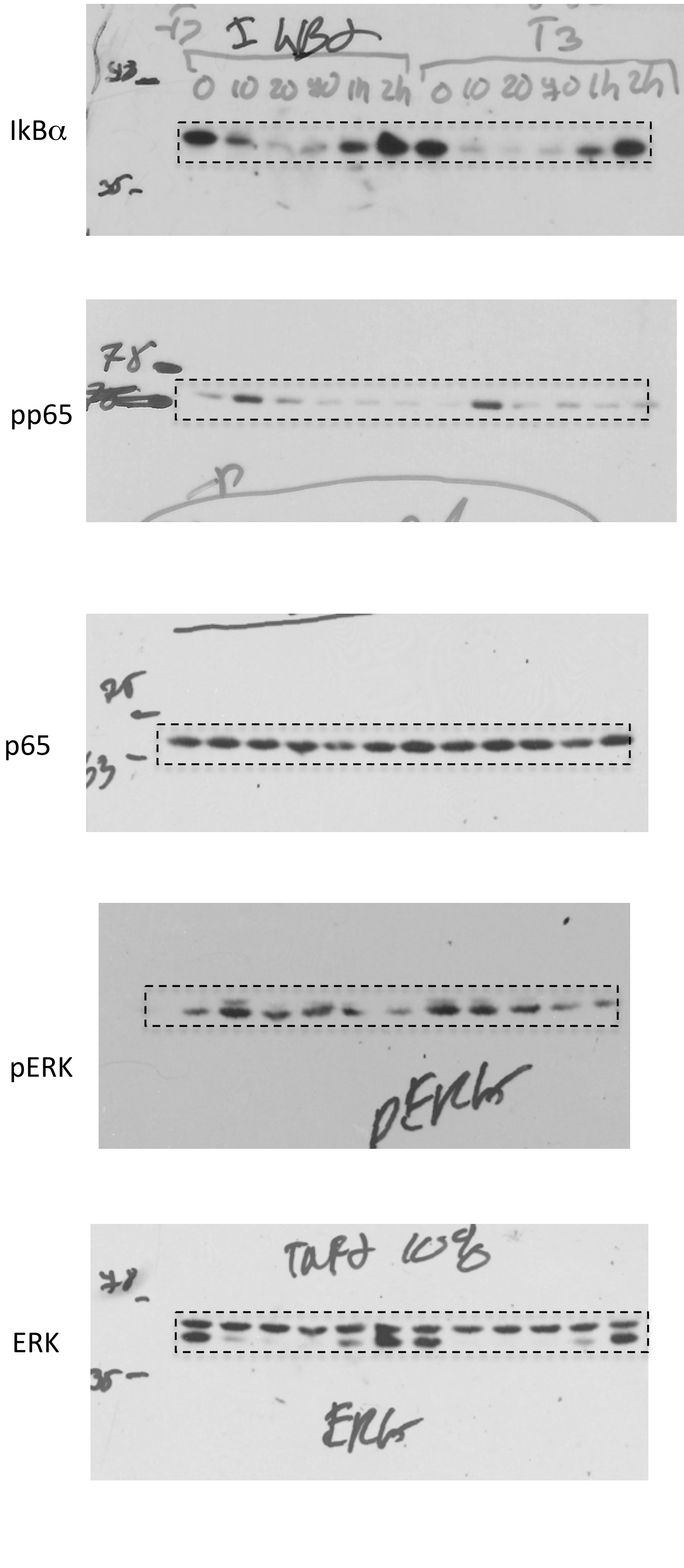

FIGURE 7A (primary macrophages)

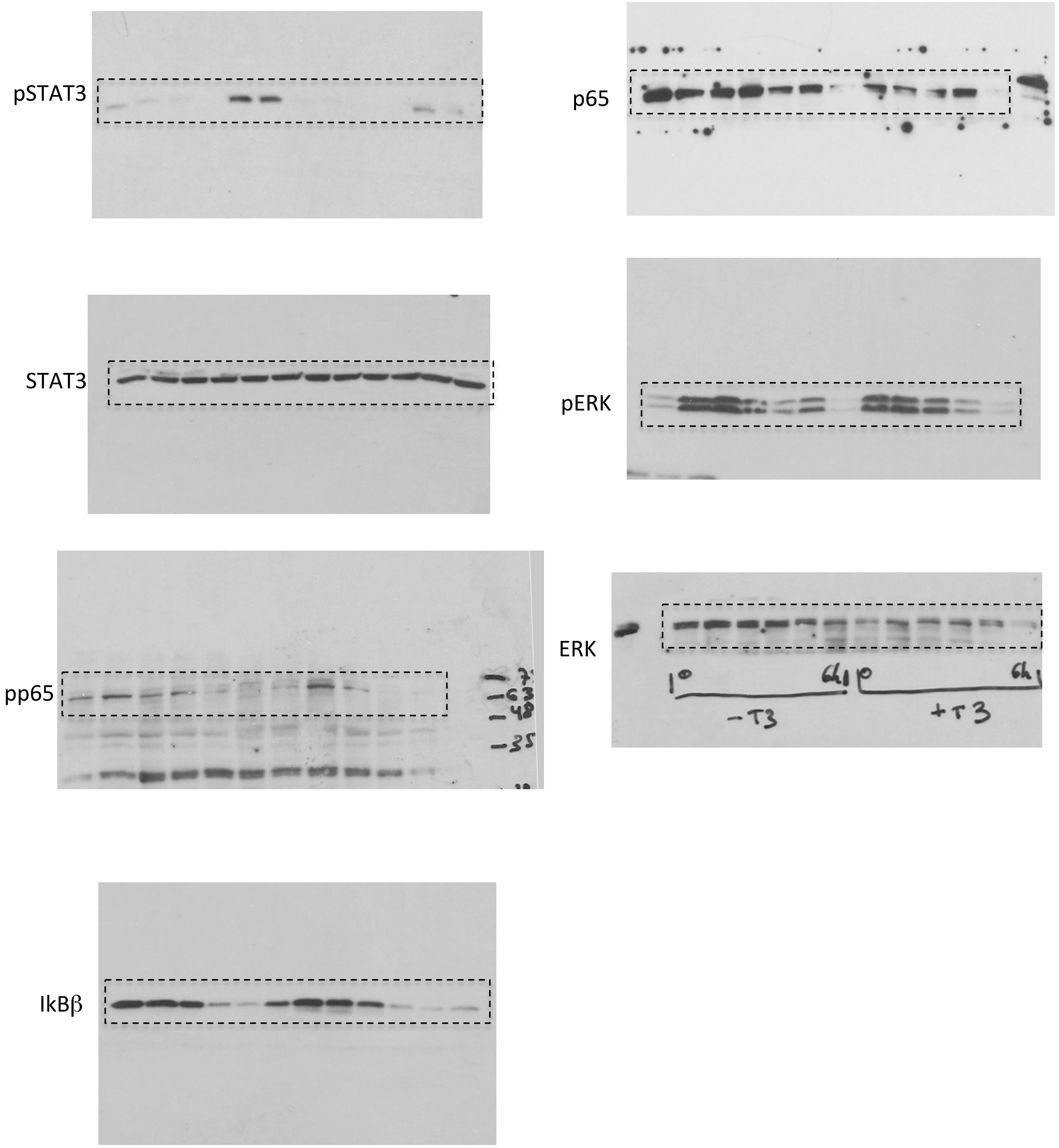

FIGURE 7ª (RAW264.7)

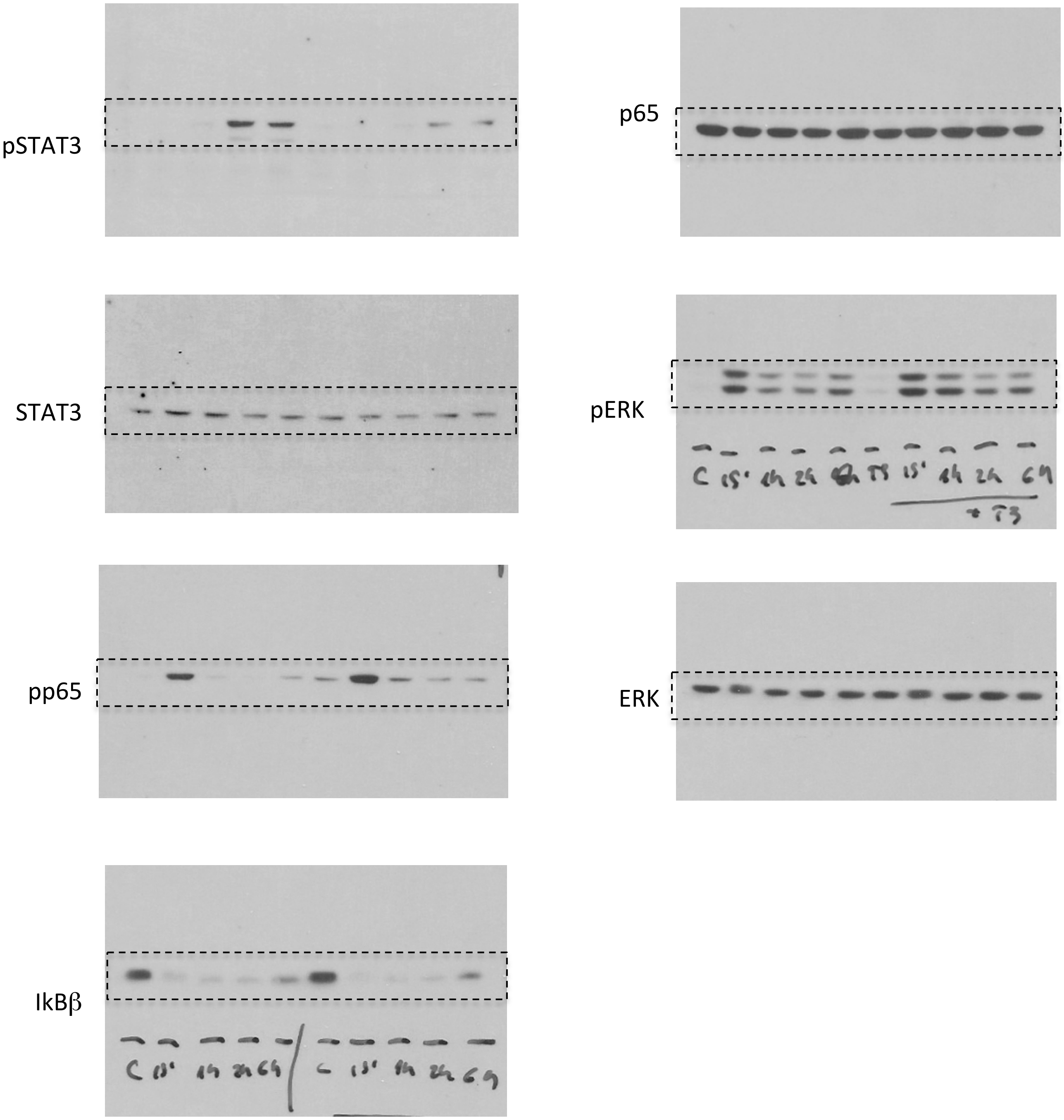

FIGURE S7 (IL-6)

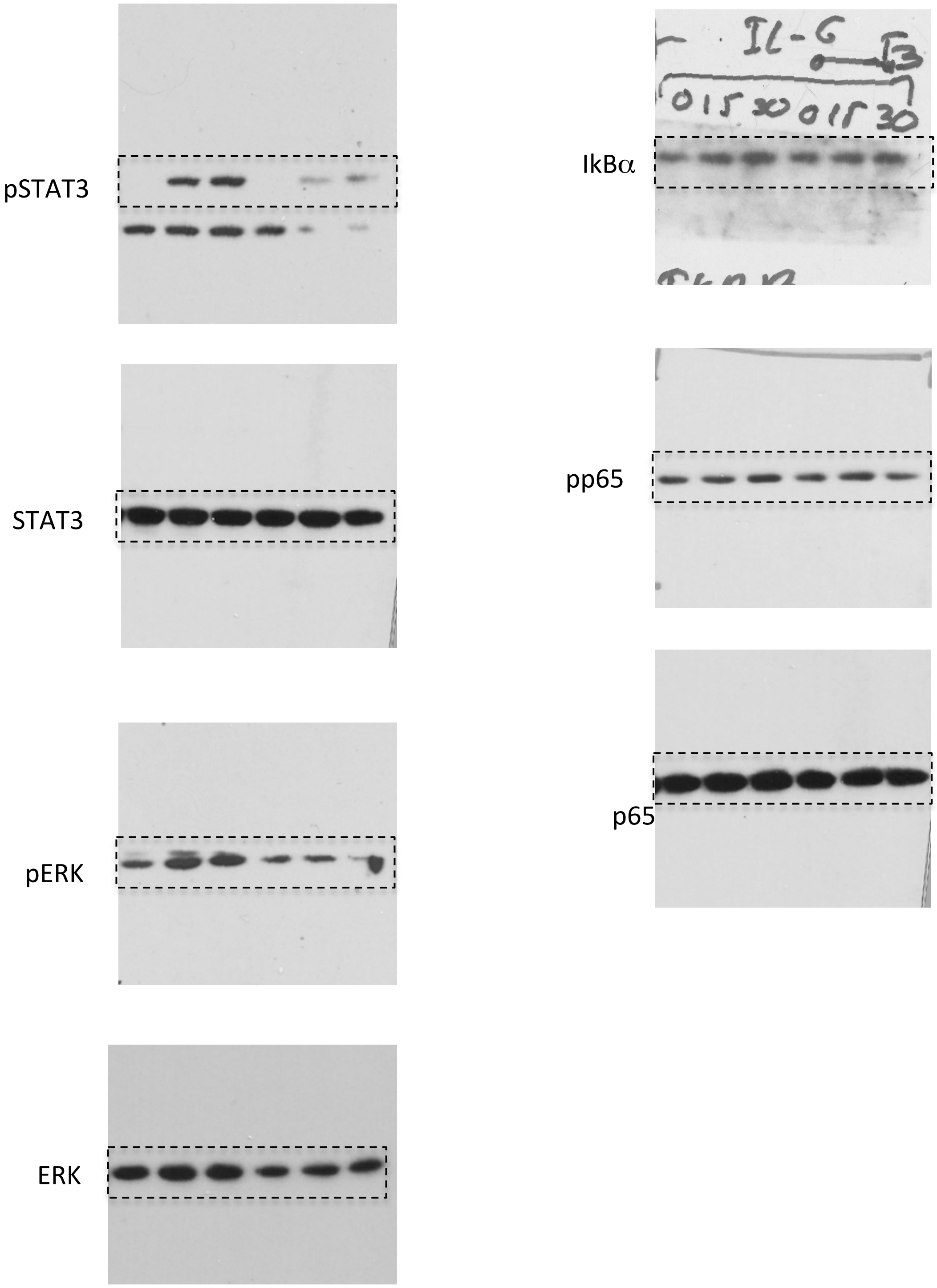

FIGURE S7 (IL-6)

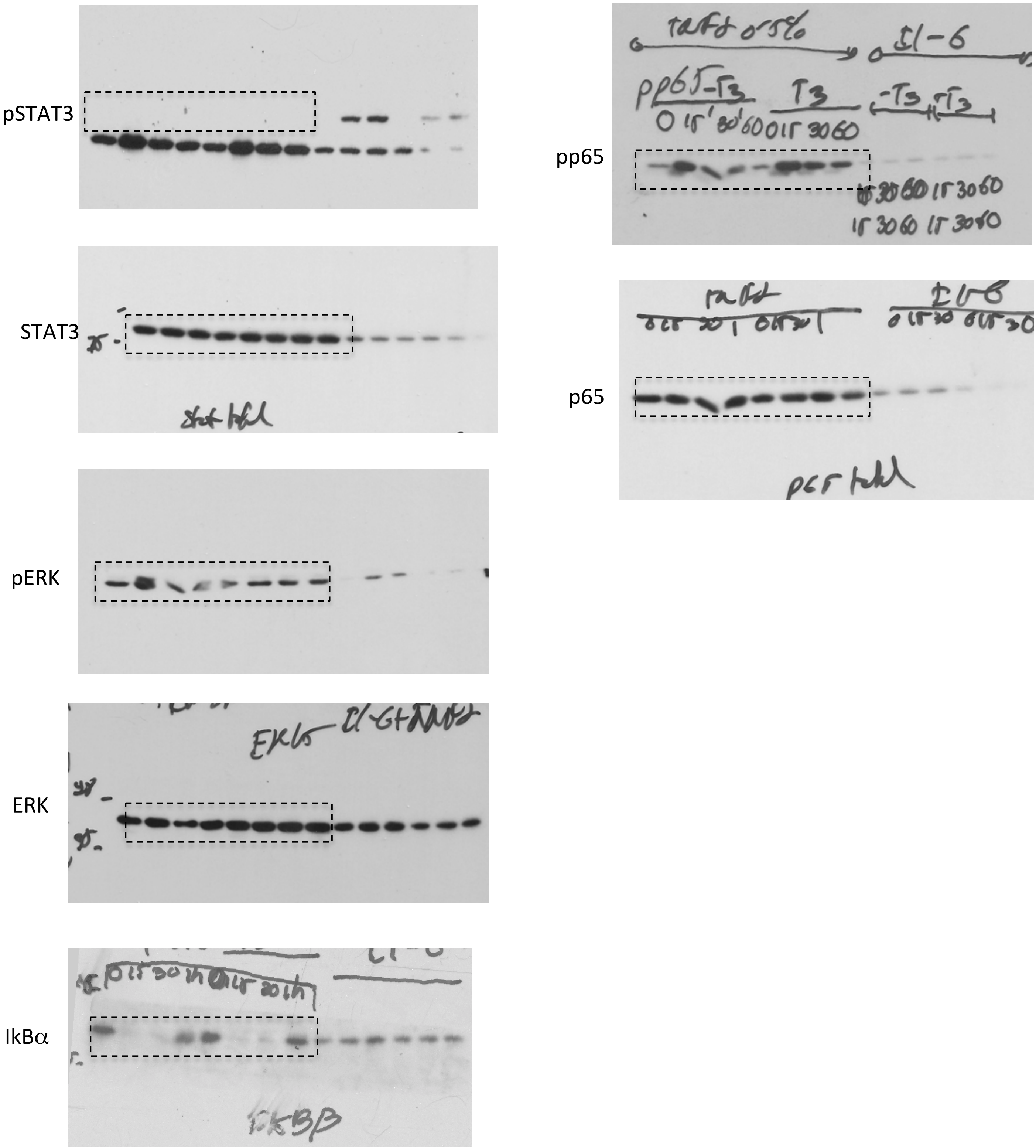

FIGURE S8

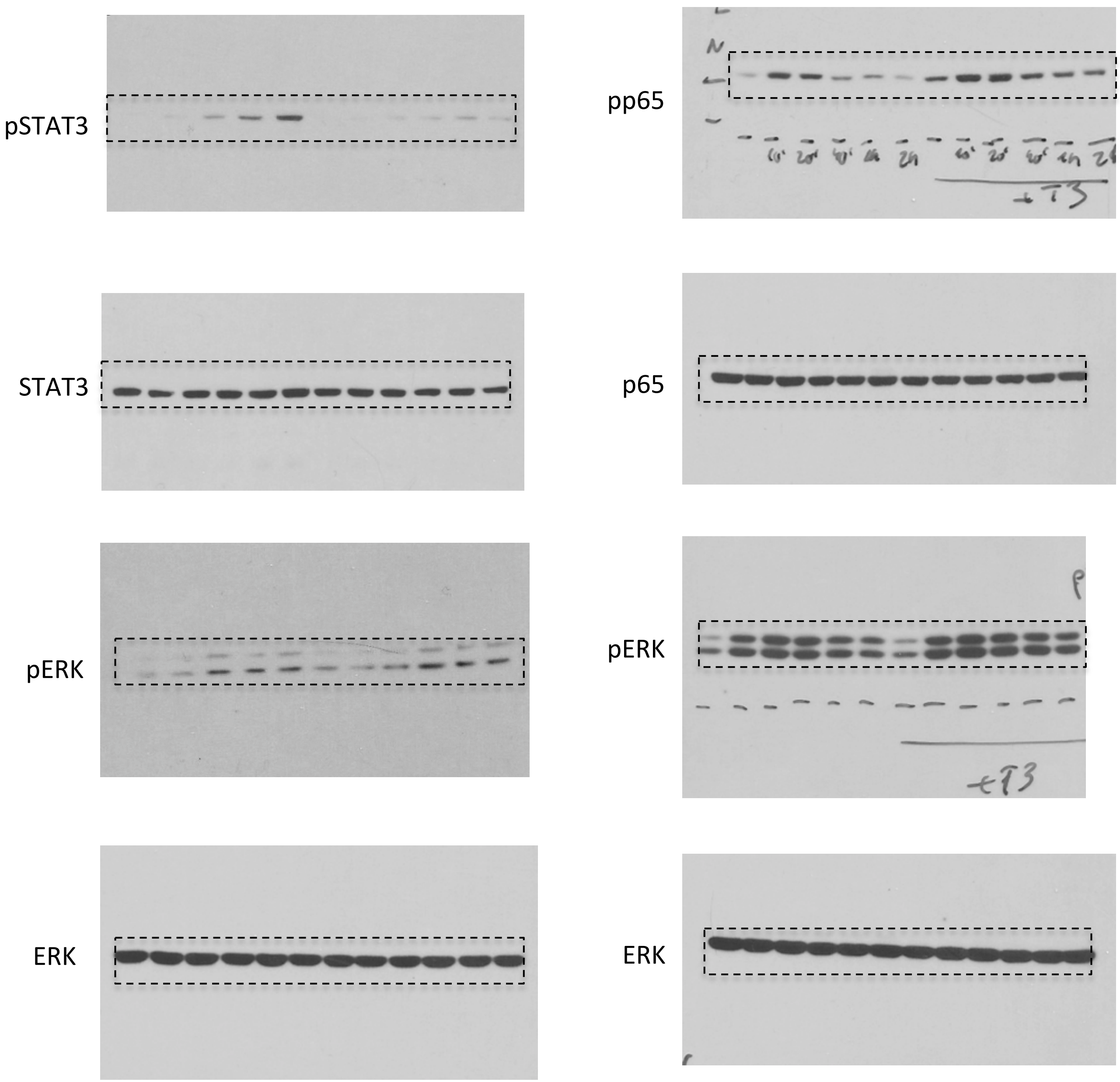

Supplement: Supplementary Information [file srep30990-s1.pdf]
